# Langevin equation in complex media and anomalous diffusion

**DOI:** 10.1098/rsif.2018.0282

**Published:** 2018-08-29

**Authors:** Silvia Vitali, Vittoria Sposini, Oleksii Sliusarenko, Paolo Paradisi, Gastone Castellani, Gianni Pagnini

**Affiliations:** 1 Department of Physics and Astronomy, Bologna University, Viale Berti Pichat 6/2, 40126 Bologna, Italy; 2 Institute for Physics and Astronomy, University of Potsdam, Karl-Liebknecht-Strasse 24/25, 14476 Potsdam-Golm, Germany; 3 BCAM–Basque Center for Applied Mathematics, Alameda de Mazarredo 14, 48009 Bilbao, Basque Country, Spain; 4 ISTI–CNR, Institute of Information Science and Technologies ‘A. Faedo’ (Consiglio Nazionale delle Ricerche), Via Moruzzi 1, 56124 Pisa, Italy; 5 Ikerbasque–Basque Foundation for Science, Calle de María Díaz de Haro 3, 48013 Bilbao, Basque Country, Spain

**Keywords:** anomalous diffusion, heterogeneous media, biological transport, Gaussian processes, space–time fractional diffusion equation, fractional Brownian motion

## Abstract

The problem of biological motion is a very intriguing and topical issue. Many efforts are being focused on the development of novel modelling approaches for the description of anomalous diffusion in biological systems, such as the very complex and heterogeneous cell environment. Nevertheless, many questions are still open, such as the joint manifestation of statistical features in agreement with different models that can also be somewhat alternative to each other, e.g. continuous time random walk and fractional Brownian motion. To overcome these limitations, we propose a stochastic diffusion model with additive noise and linear friction force (linear Langevin equation), thus involving the explicit modelling of velocity dynamics. The complexity of the medium is parametrized via a population of intensity parameters (relaxation time and diffusivity of velocity), thus introducing an additional randomness, in addition to white noise, in the particle's dynamics. We prove that, for proper distributions of these parameters, we can get both Gaussian anomalous diffusion, fractional diffusion and its generalizations.

## 1. Introduction

The very rich dynamics of biosystem movements have been attracting the interest of many researchers in the field of statistical physics and complexity for its inherent temporal and spatial multi-scale character. Further, new techniques allowed tracking the motion of large biomolecules in the cell with great temporal and spatial accuracy, both *in vivo* and *in vitro* [1–3]. Two main transport mechanisms were identified: (i) passive motion, determined by cytoplasm crowding and (ii) active transport, given by the presence of molecular motors carrying biomolecules along filaments and microtubules (cytoskeleton) [4–7]. Diffusion processes have been used to describe many biological phenomena such as molecular motion through the cellular membrane [8–11], DNA motility within the cell nucleus [6], chromosome dynamics and motility on fractal DNA globules [12], motion of mRNA molecules in *Escherichia coli* bacteria [5] and of lipid granules in yeast cells [4].

Standard or normal diffusive (Brownian) motion is uniquely described by the Wiener process [13] and is associated with a Gaussian probability density function (PDF) of displacements and linear time dependence of the mean square displacement (MSD). It is well-known that normal diffusion emerges in the long-time limit *t* ≫ *τ*_c_ when the correlation timescale *τ*_c_ is finite and non-zero [14] (see section 1 of the electronic supplementary material for details). However, diffusion in biosystems is often non-standard, with non-Gaussian PDF of displacements and nonlinear time dependence of MSD:1.1
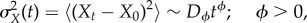
where *X*(*t*) is the position. This is known as *anomalous diffusion*, distinguished in slow *subdiffusion* (*ϕ* < 1) and fast *superdiffusion* (*ϕ* > 1). Normal diffusion is recovered for *ϕ* = 1.

The general condition for anomalous diffusion to occur is to have a zero or infinite *τ*_c_ [14] and, precisely:

— *superdiffusion*:1.2
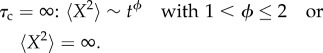
— *subdiffusion*:1.3
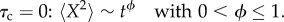

(see section 1 of the electronic supplementary material for a detailed discussion about this point).

Both subdiffusion and superdiffusion have been found in cell transport, the first one usually being related to passive motion and the latter one to active motion (see, e.g. [4,5,15,16] for subdiffusion, and [6,7,17,18] for superdiffusion).

At variance with normal diffusion different physical/biological conditions can originate anomalous diffusion [19,20] and several models and interpretations have been proposed in the recent literature [1,3,21,22]. Widely investigated models of anomalous diffusion are continuous time random walk (CTRW) [20] and fractional Brownian motion (FBM) [23], both models sharing the same anomalous diffusive scaling of equation (1.1). Many authors have compared these models with each other and with data, essentially finding some features to be satisfied by the CTRW (weak ergodicity breaking and ageing) [21,24,25] and other ones by the FBM (e.g. the *p*-variation index [26–28]). Despite the efforts of many research groups, an exhaustive model explaining all the statistical features of experimental data does not yet exist and the research is recently focusing on alternative approaches, such as heterogeneous diffusivity processes (HDPs) [29–33] or other similar approaches based on fluctuations of some dynamical parameter, e.g. fluctuating friction governed by a stochastic differential equation [34–36], mass of a Brownian-like particle randomly fluctuating in the course of time [37].

All these approaches can be linked to superstatistics [38,39], whose main idea is that of a complex inhomogeneous environment divided into cells, each one characterized by a nearly uniform value of some intensive parameters. Then, a Brownian test particle experiences parameter fluctuations during a cell-to-cell transition [39]. In general, superstatistics is successful to model: turbulent dispersion (energy dissipation fluctuations) [38], renewal critical events in intermittent systems [40,41] and, for different distributions of the fluctuating intensive quantities, different effective statistical mechanics can be derived [39], e.g. Tsallis statistics with *χ*^2^-distribution [38]. Diffusing diffusivity models (DDMs), with position diffusivity governed by a stochastic differential equation, have been recently proposed [31] and are attracting the interest of many authors as they represent an important attempt to go beyond superstatistics [33,42–44].

In this framework, we propose a modelling approach to anomalous diffusion inspired by the constructive approach used to derive Schneider grey noise, grey Brownian motion (gBM) [45,46] and generalized gBM (ggBM) [47–52] (see section 5 of the electronic supplementary material for a brief survey about grey noise, gBM and ggBM). Such processes emerge to be equivalent to the product of the FBM *B*_*H*_(*t*) with an independent positive random variable λ, i.e. the amplitude associated with each single trajectory can change from one trajectory to another one (*H* is the self-similarity Hurst exponent). When the amplitude PDF is the Mainardi distribution *M*_*β*_(λ) with properly chosen scaling *β* (depending on the FBM scaling *H*) [53–55], grey noise is a stochastic solution of the time fractional diffusion equation (TFDE) [56–58], i.e. the gBM-PDF *P*(*x*, *t*) is a solution of the TFDE (see section 6 of the electronic supplementary material for a brief survey about the Mainardi function). The ggBM generalizes gBM by considering independent scaling parameters *β* and *H* and it was recently recognized to be a stochastic solution of the Erdélyi–Kober fractional diffusion equation (EKFDE) [59]. A further extension of the ggBM is given by the process introduced in [60], where the amplitude distribution is generalized to a combination of Lévy distributions by imposing the ggBM-PDF to be compatible with the space–time fractional diffusion equation (STFDE) [56–58,61]. Interestingly, ggBM can also describe non-stationary and aging behaviours. The potential applications of ggBM to biological transport were recently discussed in [62], where the ggBM compatible with EKFDE was investigated by means of several statistical indices commonly used in the analysis of particle tracking data. The authors showed that the ggBM approach accounts for the weak ergodicity breaking and ageing (CTRW) and, at the same time, for the *p*-variation test (FBM). A DDM and a ggBm-like model (namely a randomly scaled Gaussian process) with random position diffusivity governed by the same stochastic equation have been recently compared each other [33]. However, the physical interpretation of the ggBM approach based on the FBM is not completely clear. Further, potential applications to transport in a viscous fluid needs to include at least the effect of viscosity.

In order to include the effect of viscosity, we describe the development of a model similar to the original ggBM, but with a friction–diffusion process instead of a Gaussian noise, thus involving an explicit modelling of the system's dynamics by substituting the FBM, used to built the ggBM, with the stochastic process resulting from the Langevin equation for the particle velocity. In particular, we use a Langevin equation with a linear viscous term (Stokes drag) and an additive white Gaussian noise, also known as the Ornstein–Uhlenbeck (OU) process [13]. The system's complexity is described by proper random fluctuations of the parameters in the velocity Langevin equation: relaxation time, related to friction; velocity diffusivity, related to noise intensity. It is worth noting that the medium is here composed of the underlying fluid substrate and of the particle ensemble. Medium complexity is then not mimicked by random temporal fluctuations, but described by inter-particle fluctuations of parameters and, thus, by proper time-independent statistical distributions that characterize the complex medium. In the next sections, we show that this assumption allows anomalous diffusion to be obtained if proper parameter distributions are chosen. In this sense, this model also generalizes the approach of HDPs as it also accounts for the heterogeneity of the friction parameter, thus including the effect of relaxation due to viscosity that, in other HDPs, is completely neglected. In this work, we focus on superdiffusion, which is derived for the free motion of a particle by means of a general argument.

The paper is organized as follows. In §2, we introduce the randomized Langevin model for superdiffusion, based on the free motion of Brownian particles in a viscous medium. In §3, we show the results of numerical simulations. In particular, we numerically test some crucial assumptions, such as the existence of a generalized equilibrium/stationary condition in the long-time limit. In §4, we sketch some conclusions and discuss the potential applications of the proposed model. Mathematical details can be found in the electronic supplementary material.

## 2. Free particle motion and superdiffusion

Consider the following linear Langevin equation for the velocity *V* (*t*) of a particle moving in a viscous medium:2.1
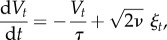
being *τ* the relaxation timescale,^1^ and *ν* the velocity diffusivity, which has dimensional units: [*ν*] = [*V*^2^]/[*T*]. The diffusivity *ν* determines the intensity of the Gaussian white noise *ξ*_*t*_. This is a random uncorrelated force:2.2
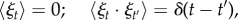
whose stochastic Itô integral is a Wiener process [13]. When *τ* and *ν* are fixed parameters, equation (2.1) is a OU process (e.g. [13]), which, together with the kinematic equation:2.3
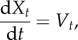
is the most simple stochastic model for the one-dimensional free motion of a particle in a viscous medium, with thermal fluctuations depicted by the white noise *ξ*_*t*_.

In the Langevin model with random parameters here proposed, single path dynamics are given by equation (2.1), but the statistical ensemble of paths is affected not only by randomness in the white noise *ξ*_*t*_, but also in the parameters *τ* and *ν*, whose randomness describes the complex medium. In order to derive the overall statistical features of *X*_*t*_ and *V*_*t*_, the computation is carried out in three steps. First we consider the averaging operation with respect to the noise term *ξ*_*t*_ and how the presence of a population for the parameters *τ* and *ν* affects some statistical properties of the process. Then we consider the average over the random parameter *τ* and we evaluate the PDF *g*(*τ*) in order to get an anomalous superdiffusive scaling. Finally, we evaluate the PDF *f*(*ν*) in order to get the distribution *P*(*x*, *t*) compatible with fractional diffusion, i.e. equal to the fundamental solutions of some class of fractional diffusion equations [59,60], or with other kinds of diffusion processes.

The averaging operation with respect to the noise term *ξ*_*t*_ gives the statistical features conditioned to the random parameters *τ* and *ν*, which result to be exactly the same as the standard OU process as shown in box 1. In particular, we are interested in the stationary correlation function conditioned to *τ* and *ν*, which reads (see equations (2.6) and (2.7), box 1):2.8
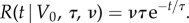
Given equation (2.8) and considering statistically independent populations of *τ* and *ν*, the stationary correlation function of the ensemble is given by:2.9
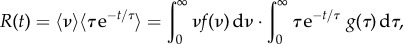
where *g*(*τ*) and *f*(*ν*) are the PDFs of the parameters *τ* and *ν*, respectively. The conditional MSD is derived from the conditional correlation function *R*(*t* | *V*_0_, *τ*, *ν*), equation (2.8), and, accordingly, the effective or global MSD (averaged over *τ* and *ν*), is derived from the global correlation function R(t), equation (2.9) (see section 1 of the electronic supplementary material). The standard OU process is recovered for 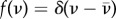 and 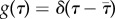, that is, when the parameters *ν* and *τ* are the same for all trajectories.

Does such stationarity correspond to an equilibrium condition? An equilibrium state is defined by the equilibrium velocity distribution, which is independent of the initial conditions and it is reached by the system after a transient time. When equilibrium is reached, the process becomes stationary: the non-stationary term of the correlation function becomes negligible and only the stationary correlation given in equation (2.8) survives. The decay of the non-stationary correlation term corresponds rigorously to equilibrium in the standard OU process with fixed *τ* and *ν* as shown in box 1. However, it is not straightforward that this feature also extends to the Langevin equation with random parameters, equation (2.1).

It is worth noting that the average of the *conditional stationary velocity variance* (equation (2.7), box 1) over *τ* and *ν* gives:2.10
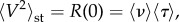
which resembles an equilibrium condition extending that of the standard OU process, by considering the mean values of *τ* and *ν*. This condition cannot be assumed *a priori*, but, if equilibrium exists, it surely needs a stationary assumption, so that, in the following, we assume that, in the long-time regime *t*_1_, *t*_2_ ≫ 〈*τ*〉, the stationary state defined by equation (2.10) is reached within a good approximation. Consequently, in this model we consider an approximated stationary condition by setting to zero the non-stationary term of the correlation function in equation (2.5) (box 1). The validity of the stationary assumption and its coincidence with the emergence of an equilibrium distribution will be discussed later and verified by means of numerical simulations^2^ (see §3.2).

Box 1. **OU statistics conditioned to *τ* and *ν*.**

The statistical features conditioned to the values of *τ* and *ν* are given by the same mathematical expressions of the standard OU process [13].

Given the initial condition *V*_0_ = *V* (0), the solution for *t* ≥ 0 of equation (2.1) is given by:2.4
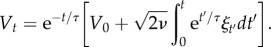

This solution can be exploited to derive the *conditional velocity correlation function*, where the average is here made over the noise *ξ*_*t*_:2.5
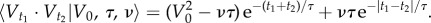

The conditional dependence of the average on the initial velocity *V*_0_ and on the parameters *τ* and *ν* has been explicitly written. The choice of the initial velocity distribution affects the way the system relaxes to the equilibrium condition, but not the equilibrium condition itself. The correlation function includes two terms: the first one is the non-stationary transient associated with the memory of the initial condition *V*_0_, while the second one is the stationary component depending only on the time lag between *t*_1_ and *t*_2_. In the long time limit *t*_1_, *t*_2_ ≫ *τ*, the first term becomes negligible, thus giving the *conditional stationary correlation function*:2.6
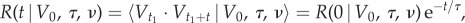

being *t* = |*t*_2_ − *t*_1_| the time lag and:2.7
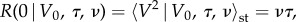

the *conditional stationary velocity variance*, which results to be independent of time *t*_1_ and of the initial velocity *V*_0_.

The correlation function *R*(*t*) defined in equation (2.9) and the PDF *g*(*τ*) must satisfy a list of features to describe superdiffusion, i.e. *σ*^2^_*X*_(*t*) ∼ *t*^*ϕ*^; *R*(*t*) ∼ *t*^*ϕ*−2^; 1 < *ϕ* < 2, concerning the asymptotic time scaling of the functions, normalization and finite mean conditions for the distribution of timescales *g*(*τ*) (see box 2 in electronic supplementary material).

It is worth noting that the statistical distribution of *ν* does not affect the scaling of the correlation function in equation (2.9), but it only introduces a multiplicative factor. Therefore, a constructive approach similar to that adopted to built up the ggBM [47,49,50,60] can be applied to our model, randomness of *τ* determining the anomalous diffusion scaling and that of *ν* the non-Gaussianity of both velocity and position distributions.

Regarding the PDF *g*(*τ*), the following:2.11
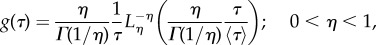
indeed satisfies all the required constrains (i–iv) listed in the electronic supplementary material (box 2, proofs in section 2). We stress that the choice of *g*(*τ*) is not arbitrary, but addressed (not derived) by the required constrains listed in box 2 of the electronic supplementary material.

In the above expression, *g*(*τ*) depends on the parameter *η*, which is the index of the Lévy stable, unilateral PDF *L*^−*η*^_*η*_, and on the mean relaxation timescale 〈*τ*〉. With the above choice, we get the following asymptotic behaviour for the stationary correlation function, conditioned to *ν*, when 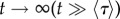 (see section 2 of the electronic supplementary material for details):2.12
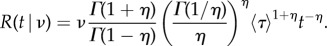
By applying equation (3, electronic supplementary material), we get the (superdiffusive) scaling for the MSD: *σ*^2^_*X*_(*t*|*ν*)∝*t*^*ϕ*^ with 1 < *ϕ* = 2 − *η* < 2.

Note that the calculations are here made under the assumption of the approximated stationary condition discussed previously. In this regime, *X*(*t*) is exactly a Gaussian variable, as it can be reduced to a sum, over time, of almost independent Gaussian distributed velocity increments. Equation (3) (or, equivalently, equation (4)) in the electronic supplementary material is essentially a sum of variances of Gaussian distributed variables, so that the overall effect of *g*(*τ*) is the emergence of a Gaussian variable with the anomalous, nonlinear, scaling of the variance given in equation (1.2).^3^ The resulting PDF of *X*_*t*_ conditioned to *ν* is then given by the following Gaussian law:2.13
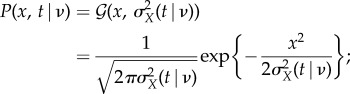
2.14
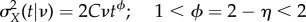
2.15
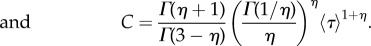


The conditional dependence of 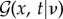 on *ν* is clearly included in *σ*^2^_*X*_(*t*|*ν*). The one-time PDF of the diffusion variable *X*_*t*_ is given by the application of the conditional probability formula:2.16
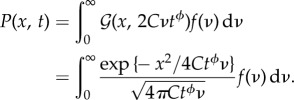
This relationship is formally similar to eqn (3.9) of [60]. Thus, comparing with this same equation and after some algebraic manipulation, equation (2.16) can be generalized to the following general form by including the scaling exponent *ϕ*:2.17
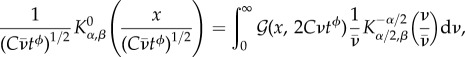
with 1 < *ϕ* = 2 − *η* < 2, 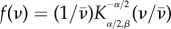 and *C* = *C*(*η*, 〈*τ*〉) given by equation (2.15). The reference scale 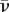 is needed to give the proper physical dimensions to the random velocity diffusivity *ν*. As we consider only symmetric diffusion, *θ* = 0, the general range of parameters *α* and *β* is given by2.18
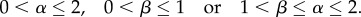
equation (2.17) is, in general, driven by three scaling indices: (i) *α* and *β*, which are related to the shape of the distribution and (ii) *ϕ*, i.e. the anomalous superdiffusive scaling of the MSD, related to the scaling exponent *η* of the correlation function *R*(*t*): *ϕ* = 2 − *η*, 0 < *η* < 1. The fundamental solution of the STFDE (section 7, electronic supplementary material), that is of particular interest for applications, is obtained with the choice of parameters: *ϕ* = 2*β*/*α*; 1 < *ϕ* < 2. Interestingly, when *ϕ* ≠ 2*β*/*α*, equation (2.17) describes a *generalized space–time fractional diffusion* that is not compatible with the STFDE.

## 3. Numerical simulations

### 3.1. Simulation set-up

In this section, we carry out numerical simulations of the superdiffusive model given by equations (2.1)–(2.3) with random *τ* and *ν*, both to compare with analytical results and to verify the accuracy of our assumptions. A total of 10 000 stochastic trajectories are computed for each simulation. To this goal, a statistical sample of 10 000 couples (*τ*,*ν*) is firstly extracted by the respective distributions, each couple being associated with one trajectory in the simulated ensemble. In all simulations, the following values are chosen: 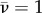; initial conditions *X*_0_ = 0 and *V*_0_ = 0 for all trajectories; total simulation *T*_sim_ = 10^3^〈*τ*〉.

Regarding the sampled populations of *ν* we consider three different distributions *f*(*ν*), corresponding to different kinds of anomalous diffusion:

(1) *Gaussian anomalous diffusion with long-range correlations*: a fixed value of *ν* is chosen to be equal for all trajectories. This is a reduced model, whose 1-time PDF is given by equations (2.13)–(2.15) and, for long time lags, the stationary correlation function is given by equation (2.12) with 0 < *η* < 1. The only random parameter labelling the trajectories is the correlation time *τ*. It is interesting to note that this model belongs to the class of Gaussian stochastic processes with stationary increments and long-range correlations, thus sharing the same basic features of FBM, but within a completely different physical framework.
(2) *Erdélyi–Kober fractional diffusion and Mainardi distribution* [51,59]: (parameter range: *α* = 2, 0 < *β* < 1, 1 < *ϕ* < 2)3.1
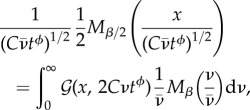
being *M*_*β*/2_/2 = *K*^0^_2,*β*_; *M*_*β*_ = *K*^−1^_1,*β*_. This is the solution of a fractional diffusion equation with Erdélyi–Kober fractional derivative in time [51,59].
  For *ϕ* = *β* the solution of the TFDE is recovered, i.e. equation (89) of the electronic supplementary material with *α* = 2. In this case, the mean velocity diffusivity 〈*ν*〉 is finite and can be computed by applying the formula for the moments of *M*_*β*_ [58]:3.2
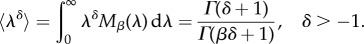
Thus:3.3
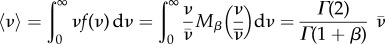
(3) *Generalized space fractional diffusion and extremal Lévy distributions*: (parameter range: *β* = 1; 1 < *α* < 2; 1 < *ϕ* < 2)3.4
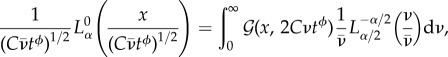
where *L*^*θ*^_*α*_ is the Lévy stable density of scaling *α* and asymmetry *θ* and *L*^0^_*α*_ = *K*^0^_*α*,1_; *L*^−*α*/2^_*α*/2_ = *K*^−*α*/2^_*α*/2,1_. The moments of both PDFs *L*^0^_*α*_ and *L*^−*α*/2^_*α*/2_ are not finite. In particular: 〈*ν*〉 = ∞. For *ϕ* = 2/*α* the solution of the space fractional diffusion equation is recovered, i.e. equation (89) of the electronic supplementary material with *β* = 1.

For the random generation of *ν*, we refer to the algorithms discussed and used in [60] (eqn (4.9) for the Lévy extremal distribution and eqn (4.6) for the Mainardi distribution), based on the Chambers–Mallows–Stuck algorithm for the generation of Lévy random variables [63,64]. The sampled population of *τ* is extracted from the PDF *g*(*τ*), equation (2.11), using the numerical random generator described in section 4 of the electronic supplementary material. It is worth noting that this algorithm is semi-analytical, that is, asymptotic solutions are used for both short and long *τ*, while in the intermediate regime the algorithm is completely numerical. The numerical scheme for the Langevin equation is described in the electronic supplementary material, section 3.

### 3.2. Discussion of numerical results

Numerical simulations have been carried out for different values of scaling parameters and show qualitatively good agreement with analytical results for both ensemble averaged MSD *σ*^2^_*X*_(*t*) and PDF *P*(*x*, *t*). The goodness of comparison decreases as the parameters get closer to the extremal allowed values of the scaling parameters that are more far from standard and/or Markovian diffusion (i.e. *η* = 1, *α* = 2, *β* = 1).

It is important to notice that, while the random generator of *ν* does not essentially determine any criticality in the numerical algorithm, the role of the parameter *τ* in the numerical implementation of the model is much more delicate. This aspect is strictly related to the equilibrium properties of single trajectories and of the overall system. In fact, the derivation of our model is based on the assumption of an equilibrium/stationary condition for all the sample paths in the statistical ensemble. This condition is exactly true only for *t* = ∞, while, for whatever finite time *t*, is clearly well approximated only for those trajectories satisfying the condition *τ* < *t*. Conversely, due to the slow decaying power-law tail in the *g*(*τ*) distribution, relaxation times *τ* much longer than 〈*τ*〉 have non-negligible probabilistic weights. Thus, 〈*τ*〉 does not really characterize the relaxation/correlation time of all stochastic trajectories, each one experiencing its own timescale to reach the equilibrium/stationary condition.

Then, two crucial aspects need to be verified: does an equilibrium condition exist? Is the timescale to reach such an equilibrium finite?

The working hypothesis to be checked is that, despite the inverse power-law tail in *g*(*τ*), the statistical weights of sufficiently large *τ* are negligible enough to get a global equilibrium condition in the range *t* ≫ 〈*τ*〉. This is a crucial aspect regarding the self-consistency of the model with respect to the existence of a global stationary condition and, last but not least, the comparison with experimental data.

**Figure 1.**
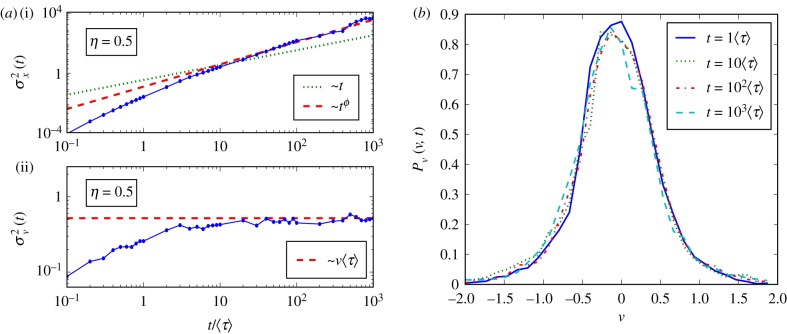
Superdiffusion with *η* = 0.5 (*ϕ* = 2 − *η* = 1.5, 〈*τ*〉 = 0.52) and fixed *ν* = 1 (Gaussian case). (*a*) MSD of velocity (ii) and position (i); (*b*) velocity (Gaussian) PDF *P*(*x*, *t*) at different times. (Online version in colour.)

The numerical simulations proved that a (global) stationary state indeed exists and that the equilibrium condition and the expected anomalous diffusion regime in the MSD are reached for times sufficiently larger than 〈*τ*〉. In figure 1, we show the results for the simulation of a statistical sample of 10 000 trajectories with *η* = 0.5 and fixed *ν* = 1 (Gaussian case). From panel (*a*(ii)) and panel (*b*), it is clear that the system reaches the stationary state within a time of the order *t* ≈ 10〈*τ*〉 or less, which is the time the particle needs to reach the theoretical stationary velocity variance 〈*V*^2^〉_st_ = 〈*ν*〉〈*τ*〉 (*a*(ii)) and the long-time diffusive scaling *ϕ* = 2 − *η* = 1.5 (*a*(i)). From panel (*b*), it is clear that velocity fluctuations reached a stationary/equilibrium condition. This characteristic time depends on *η* as it decreases while *η* increases. This feature is due to *g*(*τ*) that, for *η* approaching 1, becomes more and more peaked tending towards a Dirac *δ* function. For *η* = 1, a unique value of *τ* is chosen for all particles, so that the relaxation time of the whole system becomes *τ* itself and we fall back into standard diffusion. Thus, numerical simulations show that the stationary condition is reached at reasonable (i.e. not too much large) times. This is a good indication that the model can compare well with experimental data, anomalous diffusion emerging in a given temporal range that is neither too short nor too long. This is true for values of scaling indices that are not too close to extremes of the definition interval (e.g. *β* far from 0), except those extremal values corresponding to time and space locality, i.e. standard diffusion and/or Markovian processes.

**Figure 2.**
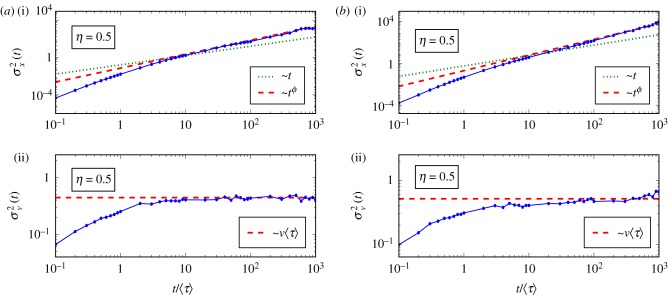
Superdiffusion with *η* = 0.5 (*ϕ* = 2 − *η* = 1.5, 〈*τ*〉 = 0.52) and fixed *ν* = 1 (Gaussian case). MSD of velocity (*a*(ii),*b*(ii)) and position (*a*(i),*b*(i)). (*a*) Sampled set with 〈*τ*〉 = 0.44 and *τ*_max_ = 75.2. (*b*) Sampled set with 〈*τ*〉 = 0.66 and *τ*_max_ = 1580.7. (Online version in colour.)

In the case of inverse power-law tails, different statistical samples extracted from the distribution *g*(*τ*) can have quite different statistics (e.g. different 〈*τ*〉). Owing to the slow power-law decay and the unavoidable finiteness of the statistical sample, the maximum value *τ*_max_ can also vary significantly among different samples. Numerical simulations for five different sampled sets of *τ* are carried out with 〈*τ*〉 = 0.52, 0.44, 0.5, 0.46, 0.66 and *τ*_max_ = 279.2, 75.2, 91.9, 200.4, 1580.7. The simulations are found to be well comparable with each other. This can be seen in figure 2, where we compare the two sampled sets of *τ* having the minimum and maximum values of *τ*_max_ (Gaussian model). Even if these values are different by orders of magnitude (from 75.2 to 1580.7), the dependence on *τ*_max_ is weak, as the time to reach stationarity changes from about 10–30 to 60–80 (see the velocity variances in *a*(ii),*b*(ii)). Further, the time to reach the stationary state does not change when comparing the Gaussian model with non-Gaussian ones (random *ν*).

**Figure 3.**
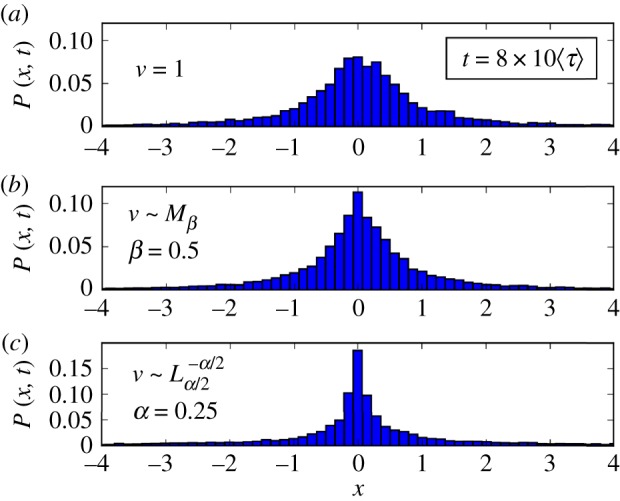
Superdiffusion with *η* = 0.5 (*ϕ* = 2 − *η* = 1.5, 〈*τ*〉 = 0.52). Comparison of PDFs *P*(*x*, *t*) for different distributions *f*(*ν*). (*a*) *ν* fixed, i.e. 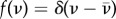 with 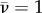 (Gaussian case); (*b*) *M*_*β*_(*μ*) distribution, *β* = 0.5 (Erdélyi–Kober fractional diffusion); (*c*) *L*^−*α*/2^_*α*/2_ distribution, *α* = 0.5 (generalized space fractional diffusion). (Online version in colour.)

**Figure 4.**
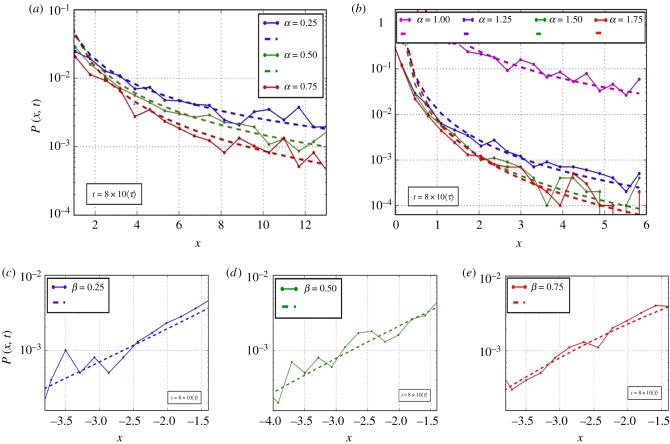
Superdiffusion with *η* = 0.5 (*ϕ* = 2 − *η* = 1.5, 〈*τ*〉 = 0.52). Comparison of analytical and numerical position PDFs *P*(*x*, *t*) in the asymptotic regime. (*a*,*b*) Different values of the scaling index *α* (*L*^−*α*/2^_*α*/2_, generalized space fractional equation); (*c*–*e*) different values of the scaling index *β* (*M*_*β*_, Erdélyi–Kober fractional diffusion). (Online version in colour.)

Figure 3 qualitatively shows the changes in the shape of the position PDF *P*(*x*, *t*) due to the *ν* randomization. Panel (*a*) displays a typical Gaussian shape. Finally, in figure 4 we compare the asymptotic tails of analytical solutions for the position PDF *P*(*x*, *t*) with the corresponding histograms computed from numerical simulations. The comparison, carried out for *η* = 0.5, show a good agreement for all the values of *α* and *β* used. Similar agreement was seen in simulations, not shown here, that were carried out for *η* = 0.25 and *η* = 0.75.

## 4. Concluding remarks

We have introduced and discussed a novel modelling approach based on a linear Langevin equation (friction–diffusion process) driven by a population of two parameters: relaxation time *τ* and velocity diffusivity *ν*, with distributions properly chosen to get anomalous diffusion (Gaussian or fractional). It is worth noting that both *τ* and *ν* directly characterize the velocity's dynamics and only indirectly the position dynamics. In particular, *ν* determines the diffusion properties of velocity and, for normal diffusion, its dimensional units are [*ν*] = [*V*^2^]/[*T*]. Gaussian anomalous diffusion is obtained by considering a constant velocity diffusivity and imposing the correct power-law correlation function compatible with MSD anomalous scaling. Fractional diffusion is derived by imposing the particular PDFs that are fundamental solutions of EKFDE or STFDE. Our stochastic model can also generate a *generalized fractional diffusion*, whose more general expression for the 1-time PDF is given in equation (2.17). In this PDF, the space–time scaling relationship is not related to the scaling indices defining the shape of the PDF itself, as in the fractional diffusion.

At variance with other HDPs, the inclusion of viscosity in our model allows us to include the effect of relaxation. The distribution of relaxation times *τ* is then a crucial property that is here derived by imposing the emergence of anomalous diffusion, retaining at the same time the Gaussianity and stationarity of velocity increments.

Another interesting aspect is the weak ergodicity breaking established in biological motion data [21,24,65] and defined by the inequality of ensemble and time averaged MSD in anomalous diffusion processes. In particular, even if the ensemble averaged MSD is given by equation (1.1), the time averaged MSD depends linearly on the time lag. In the model here proposed, the single trajectory is driven by the linear Langevin equation describing the OU process, which is characterized by the crossover between a short-time ballistic diffusion: *σ*^2^_*X*_(*t*) ∼ *t*^2^; and a long-time standard (Gaussian) diffusion: *σ*^2^_*X*_(*t*) ∼ *t*. Thus, the single trajectory naturally follows a standard diffusion law in the long-time limit. The non-ergodic behaviour is modelled by considering the randomness of physical properties and, in particular, relaxation time and velocity diffusivity, the first one driving the drift (linear viscous drag) and the second one driving the noise, respectively.

An important observation regarding the comparison between our ggBM-like modelling approach and other similar approaches is in order. All these heterogeneity-based models attempt to describe the role of heterogeneity in triggering the emergence of long-range correlations and anomalous diffusion. However, superstatistics and other models (fluctuating friction or mass, DDMs) mimic heterogeneity through the temporal stochastic dynamics or modulation of some parameters driving the particle's dynamics. On the contrary, ggBM-like models explicitly describe the heterogeneity as inter-particle fluctuations of parameters that are responsible for long-range correlations, in agreement with approaches based on polydispersity where classical thermodynamics holds [66].

Future investigations are needed not only to better understand these last observations but also, on one side, to characterize our proposed model in terms of several statistical indicators that are commonly used in the analysis of biological motions and, on the other side, to better understand the link of the parameter distributions to the observable physical properties of the complex medium. Finally, our modelling approach can be extended to the subdiffusive case by considering a kind of trapping mechanism such as a stable fixed point.

## Supplementary Material

Supplementary Material for the paper: Vitali, Sposini, Sliusarenko, Paradisi, Castellani and Pagnini: Langevin equation in complex media and anomalous diffusion

## Endnotes

1 Given the particle mass *m* and the friction coefficient *γ*, it results: *τ* = *m*/*γ*.

2 The existence of an equilibrium distribution is actually verified by means of numerical simulations and it is also shown to coincide with the validity of equation (2.10) in the long-time regime. As a consequence, by applying the average over *τ* and *ν* to the conditional velocity correlation function (equation (2.5), box 1), we find that the first term is exactly zero when the initial velocity distribution is the equilibrium one and this proves that our model is self-consistent.

3 It is worth noting that the random superposition of Langevin equations with randomized *τ* is an example of a Gaussian process with anomalous diffusion scaling that is different from the standard (FBM).

## Data accessibility

The software for the numerical simulations were written in Python 2.7 and C++ and can be downloaded at the following website: https://gitlab.bcamath.org/opensource/lecm. The simulation runs were performed using the computational facilities of BCAM-Basque Center for Applied Mathematics.
