# Supplementary material for: Langevin equation in complex media and anomalous diffusion
Source: J R Soc Interface. 2018 Aug 29;15(145):20180282. doi: 10.1098/rsif.2018.0282 (PMC6127165; doi:10.1098/rsif.2018.0282)
Supplement: Supplementary Material for the paper: Vitali, Sposini, Sliusarenko, Paradisi, Castellani and Pagnini: Langevin equation in complex media and anomalous diffusion [file rsif20180282supp1.pdf]

Supplementary Material for *Langevin equation in complex media and anomalous diffusion* Journal of Royal Society - Interface (2018)

Silvia Vitali[a], Vittoria Sposini[b,c], Oleksii Sliusarenko[c], Paolo Paradisi[d,c,*], Gastone Castellani[a], Gianni Pagnini[c,e,**]

[a]*Department of Physics and Astronomy, Bologna University, Viale Berti Pichat 6/2, I-40126 Bologna, Italy*
[b]*Institute for Physics and Astronomy, University of Potsdam, Karl-Liebknecht-Strasse 24/25, D-14476 Potsdam-Golm, Germany*
[c]*BCAM–Basque Center for Applied Mathematics, Alameda de Mazarredo 14, E-48009 Bilbao, Basque Country, Spain*
[d]*ISTI–CNR, Institute of Information Science and Technologies "A. Faedo (Consiglio Nazionale delle Ricerche), Via Moruzzi 1, I-56124 Pisa, Italy*
[e]*Ikerbasque–Basque Foundation for Science, Calle de María Díaz de Haro 3, E-48013 Bilbao, Basque Country, Spain*

NOTE:
In the following, references to equations in the Main Text are labeled with the acronym MT. For example, Eq. (1) of the Main Text is referred to as (1,MT).

1. General condition for the emergence of anomalous diffusion

Diffusion is described through the following simple, but general stochastic equation:

$$\frac{dX_t}{dt} = V_t \tag{1}$$

being V_t a stochastic process describing a generic random fluctuating signal. Here X_t and V_t are the position and velocity of a particle moving in a random medium, respectively. For a generic, nonstationary process, the two-time Probability Density Function (PDF) $p(V_1, t_1; V_2, t_2)$ depends on both times t_1 and t_2. Similarly, the correlation function

$$\langle V_{t_1} \cdot V_{t_2} \rangle = \int V_1 \; V_2 \; p(V_1, t_1; V_2, t_2) dV_1 dV_2$$

*Corresponding author
**Corresponding author
Email addresses: paolo.paradisi@cnr.it (Paolo Paradisi), gpagnini@bcamath.org (Gianni Pagnini)

is, in general, a function of the times t_1 and t_2[1].

Now, by integrating in time the above kinematic equation (1), making the square and the ensemble average, we get the Mean Square Displacement (MSD):

$$\sigma_X^2(t) = \langle (X_t - X_0)^2 \rangle = \int_0^t dt' \int_0^t dt'' \langle V_{t'} \cdot V_{t''} \rangle \ , \qquad (2)$$

where, in order to get : $\langle X_t \rangle = X_0$, we assumed a uniform initial position X_0. In the stationary case, the two-time statistics, including the correlation function, depends only on the time lag $t = |t_1 - t_2|$, and the above formula reduces to:

$$\sigma_X^2(t) = \int_0^t dt' \int_0^t dt'' R(|t' - t''|) = 2 \int_0^t (t - s) R(s)\, ds \ , \qquad (3)$$

or, equivalently:

$$\frac{d\sigma_X^2(t)}{dt} = 2 \int_0^t R(s) ds \ . \qquad (4)$$

where $R(t) = \langle V_{t_1+t} \cdot V_{t_1} \rangle = \langle V_t \cdot V_0 \rangle$ is the stationary correlation function. Notice that these expressions have very general validity, independently of the particular statistical features of V_t.

These expressions were firstly published by Taylor in 1921 [1], which implicitly formulated the following:

Theorem (Taylor 1921)

Given the stationary correlation function $R(t)$, let us define the correlation time scale:

$$\tau_c = \int_0^\infty \frac{R(s)}{R(0)} ds \ , \quad R(0) = \langle V^2 \rangle_{\rm st} \ . \qquad (5)$$ [2]

Then, if the following condition occurs:

$$0 \neq \tau < +\infty \ , \qquad (6)$$

normal diffusion always emerges in the long-time regime:

$$t \gg \tau_c \quad \Rightarrow \quad \sigma_X^2(t) = 2 D_x t \ , \qquad (7)$$

thus defining the long-time spatial diffusivity D_x:

$$D_x := \frac{1}{2} \lim_{t \to +\infty} \frac{d\sigma_{\rm x}^2}{dt}(t) \qquad (8)$$

independently from the details of the microdynamics driving the fluctuating velocity V_t.

[1] This also means that the statistics of V_t increments: $\Delta V_{t_1,t} = V_{t_1+t} - V_{t_1}$, depend not only on the time lag t, but also on the initial time t_1

[2] Notice that the variance $\langle V^2 \rangle_{\rm st}$, being a one-time statistical feature, is a constant in the stationary case.

It is worth noting that, substituting Eq. (4) into Eq. (8) and using $R(0) = \langle V^2\rangle_{\rm st}$ (Eq. (5)), we get:

$$D_X = \tau_c \, \langle V^2\rangle_{\rm st} \; , \tag{9}$$

which is a general form of the Einstein–Smoluchovsky relation [2][3].

Taylor's theorem gives in Eq. (6) the general conditions to get normal diffusion, i.e., a linear scaling in the variance: $\langle X^2\rangle \sim t$). This result has a very general validity, independently from the statistical features of the stochastic process V_t. The theorem also establishes the regime of validity of normal diffusion, given by the asymptotic condition $t \gg \tau_c$. As a consequence, the emergence of *anomalous diffusion* is strictly connected to the failure of the assumption (6). In particular, we get two different cases:

- *Superdiffusion*:

$$\tau_c = \infty : \; \langle X^2\rangle \sim t^\phi \;\; \text{with} \;\; 1 < \phi \le 2 \;\; \text{or} \;\; \langle X^2\rangle = \infty \; . \tag{10}$$

- *Subdiffusion*:

$$\tau_c = 0 : \; \langle X^2\rangle \sim t^\phi \;\; \text{with} \;\; 0 < \phi \le 1 \; . \tag{11}$$

In order to get $\tau_c = 0$ and, thus, subdiffusion, velocity anti-correlations must emerge. This means that there exist time lags t such that $R(t) < 0$ (e.g., the anti-persistent Fractional Brownian Motion, with $H < 0.5$). Being $R(0) = \langle V^2\rangle_{\rm st} > 0$, in subdiffusion the correlation function is surely positive in the short-time regime and (i) becomes negative in the long-time regime or (ii) oscillates between positive and negative values[4].

The failure of Taylor's theorem and of condition (6) is the main guiding principle exploited here to derive stochastic models for anomalous diffusion.

1.1. Application to Fractional Brownian Motion

The Fractional Brownian Motion (FBM) $B_H(t)$ was introduced by Mandelbrot and Van Ness in their famous 1968's paper [3]. Since then, thousands of papers have been devoted to both theoretical investigations and applications of FBM (see, e.g., [4] for a review). FBM is a Gaussian process with self-similar stationary increments and long-range correlations. In formulas, FBM has the following properties:

[3] Interestingly, this relation is here derived in a very general framework, i.e., for a generic fluctuating signal V_t, with the only assumption of the existence of a stationary regime in the long-time limit. As known, the stationary condition usually emerges in correspondence of motion reaching an equilibrium state. However, the stationary condition is more general with respect to equilibrium and, for this reason, we prefer to leave the notation "st" for "stationary" instead of "eq" for "equilibrium".

[4] A correlation time scale, different from the above definition of τ_c can be sometimes introduced for subdiffusion (e.g., the time period in a harmonic correlation function), but it does not have the meaning of discriminating a long-time regime with normal diffusion from a short-time regime.

- $B_H(t)$ has stationary increments;
- $B_H(0) = 0$; $\langle B_H(t) \rangle = 0$ for $t \geq 0$;
- $\langle B_H^2(t) \rangle = t^{2H}$ for $t \geq 0$;
- $B_H(t)$ has a Gaussian distribution for $t > 0$;
- the correlation function is given by:

$$\langle B_H(t) B_H(s) \rangle = \frac{1}{2} \left\{ t^{2H} + s^{2H} - |t - s|^{2h} \right\} \tag{12}$$

The FBM increments are given by:

$$V_{\delta t}(s) = B_H(s + \delta t) - B_H(s) \ .$$

The process $V_{\delta t}(s)$ is also called *fractional Gaussian noise*[5]. Both $B_H(t)$ and $V_{\delta t}(s)$ are self-similar stocastic processes but, at variance with $B_H(t)$, the increments $V_{\delta t}(s)$ are also stationary, i.e., their statistical features do not depend on s, but only on δt. $V_{\delta t}(s)$ is a Gaussian process and is uniquely defined by the mean, variance and correlation function, which are derived from the above listed properties of FBM:

$$\langle V_{\delta t}(s) \rangle = 0 \ ; \quad \langle V_{\delta t}^2(s) \rangle = (\delta t)^{2H} \tag{13}$$

$$R(t) = \langle V_{\delta t}(s) V_{\delta t}(s + t) \rangle = \frac{1}{2} \left\{ |t + \delta t|^{2h} - 2t^{2H} + |t - \delta t|^{2H} \right\} \tag{14}$$

Then, we can say that FBM is a Gaussian process with stationary and self-similar increments $V_s(\delta t)$, while FBM is Gaussian, self-similar but not stationary. Eq. (14) also shows that, with the exception of the standard Brownian motion ($H = 1/2$), increments $V_{\delta t}(s)$ are not independent each other. Fractional Gaussian noise and FBM are exactly self-similar, i.e., they satisfy the relationship: $X(at) = a^H X(t)$, the increment $V_1(s)$ with $\delta t = 1$ is usually considered in both theoretical and experimental studies, as a generic δt can be obtained by simply rescaling the process with the self-similarity relationship. In Fig. 1 the increment correlation functions of a persistent ($H > 0.5$) and of an antipersistent ($H < 0.5$) FBM are compared. It is evident that antipersistent FBM is associated with anticorrelations, and this is the reason why subdiffusion emerges in this case.

[5] This can be considered as a kind of velocity for the FBM, even if it must be kept in mind that FBM, such as standard Brownian motion, does not have a smooth velocity. In any case, the above considerations about velocity and position and their statistical relationship can here be applied by substituting velocity with the fractional Gaussian noise, i.e., the FBM increments over a finite time step δt.

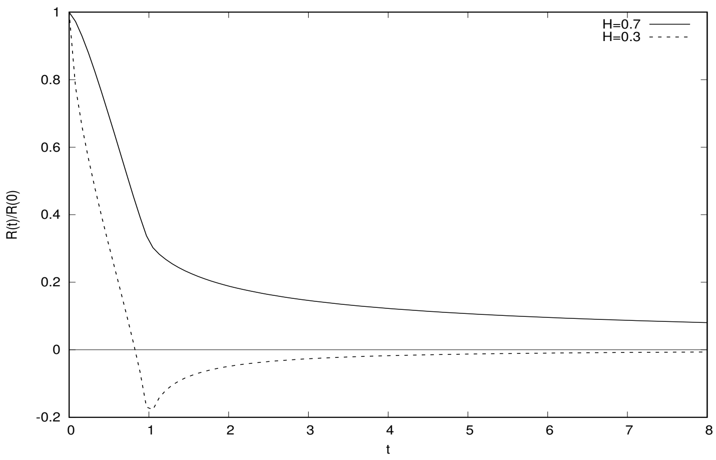

Figure 1: Autocorrelation function of FBM increments $V_1(t)$: $R(t) = \langle V_1(s)V_1(s+t)\rangle$: persistent ($H = 0.7$) vs. antipersistent ($H = 0.3$) case.

The asymptotics of the correlation function are easily obtained by rewriting it in the following way (see [4], pages 6-7):

$$R(t) = \frac{1}{2}t^{2h}h_H\left(\frac{\delta t}{t}\right) \ , \tag{15}$$

being, for $x = \delta t/t < 1$:

$$h_H\left(x\right) = (1+x)^{2H} - 2 + (1-x)^{2H} \ . \tag{16}$$

The limit $t \to \infty$ corresponds to $x \to 0$ and the Taylor expansion of $h_H\left(x\right)$ gives:

$$h_H(x) = 2H(2H-1)x^2 + O(x^4) \ , \tag{17}$$

so that [3, 4]:

$$R(t) \simeq H(2H-1)(\delta t)^2 t^{2H-2} \ . \tag{18}$$

Regarding the correlation time τ_c defined in Eq. (5), we can exploit the same asymptotic expansion used for $R(t)$. Firstly, we apply Eq. (5) to a finite time t:

$$\tau_c(t) = \int_0^t \frac{R(s)}{R(0)}ds \ , \quad R(0) = \langle V^2\rangle_{\rm st} \ , \tag{19}$$

so that: $\tau_c = \lim_{t\to\infty}\tau_c(t)$. Then, for the fractional Gaussian noise we get:

$$\tau_c(t) = \frac{\delta t}{4H+2}\left\{\left(1+\frac{t}{\delta t}\right)^{2H+1} - 2\left(\frac{t}{\delta t}\right)^{2H+1} + \left|\frac{t}{\delta t}-1\right|^{2H+1}\right\} \ . \tag{20}$$

Analogously to $R(t)$, this can be written as:

$$\tau_c(t) = \frac{\delta t}{4H+2}\left(\frac{t}{\delta t}\right)^{2H+1} h_{H+1/2}(x) \ ; \quad x = \delta t/t \ , \tag{21}$$

and, for $x < 1$, $h_{H+1/2}(x)$ is again given by Eq. (16), but with $H + 1/2$ instead of H. Then, an asymptotic formula similar to Eq. (17) can be derived:

$$h_{H+1/2}(x) = 2H(2H+1)x^2 + O(x^4) \ , \tag{22}$$

and, finally:

$$\tau_c(t) = H(\delta t)^{2-2H} t^{2H-1} \quad \text{for} \quad t \to \infty \ . \tag{23}$$

Clearly, the mathematical limit $t \to \infty$ corresponds to the physical regime $t \gg \delta t$. Exploiting the asymptotic behavior of $\tau_c(t)$ given in Eq. (23), we can now derive the values of the correlation time scale $\tau_c = \tau_c(\infty)$:

$$\tau_c = \lim_{t \to \infty} \tau_c(t) = \begin{cases} +\infty \ ; & 1/2 < H \leq 1 \ ; \\ \\ \delta t/2 < \infty \ ; & H = 1/2 \ ; \\ \\ 0 \ ; & 0 < H < 1/2 \ . \end{cases} \tag{24}$$

The three cases correspond to persistent (superdiffusive) FBM, normal Brownian motion and antipersistent (subdiffusive) FBM, respectively.

Box 2. Properties of $R(t)$ and $g(\tau)$

The use of Laplace transform, defined by the expression:

$$\widetilde{u}(s) = \mathcal{L}_{t\to s}[u(t)](s) = \int_0^\infty e^{-st} u(t) dt \,,$$

gives important information about the normalization and moments of distributions. The stationary correlation function $R(t)$ and the distribution $g(\tau)$ are related by Eq. (8,MT). For any choice of the distribution $g(\tau)$, the correlation function $R(t)$ and $g(\tau)$ must satisfy the following properties:

(i) The distribution $g(\tau)$ must be a PDF normalized to 1:

$$\widetilde{g}(0) = 1 \,,$$

which determines a constrain on the behavior of the first derivative of the correlation function:

$$\lim_{s\to+\infty} s \cdot \mathcal{L}\left[-\frac{dR(t)}{dt}\right](s) = -\frac{dR}{dt}(0_+) = \langle\nu\rangle \,.$$

(ii) The MSD is a power-law of time with superdiffusive scaling $1 < \phi < 2$ in the asymptotic long-time limit:

$$\lim_{t\to\infty} \frac{\sigma_X^2(t)}{t^\phi} = C_1 \;; \quad \lim_{s\to 0} s^{1+\phi} \cdot \widetilde{\sigma_X^2}(s) = C_2 \,. \tag{25}$$

where C_1 and C_2 are proper constants and the second asymptotic limit follows from the Tauberian theorem [5]. From Eq. (3) or Eq. (4) it results:

$$\frac{d^2\sigma_X^2(t)}{dt^2} = 2\,R(t) \;; \quad \widetilde{\sigma_X^2}(s) = \frac{2}{s^2}\widetilde{R}(s) \,,$$

we get equivalently the following expression for the stationary correlation function:

$$\lim_{t\to\infty} \frac{R(t)}{t^{\phi-2}} = C_3 \;; \quad \lim_{s\to 0} s^{1-\eta} \cdot \widetilde{R}(s) = C_4 \tag{26}$$

with $\eta = 2 - \phi$, $0 < \eta < 1$. Note that the above limits can be equivalently written as asymptotic behaviors, e.g.: $R(t) \sim t^{\phi-2}$ for $t \to \infty$, which means that the function $R(t)$ is approximated by $C_3 t^{\phi-2}$ in the long time range.

(iii) The MSD at time zero is zero:

$$\lim_{t\to\infty} \sigma_X^2(t) = 0 \,; \quad \lim_{s\to+\infty} s \cdot \widetilde{\sigma_X^2}(s) = 0$$

(iv) Furthermore being $0 < R(0) < \infty$, from Eq.(9,MT) the distribution $g(\tau)$ must have non-zero, finite mean:

$$\lim_{s\to+\infty} s \cdot \widetilde{R}(s) = R(0) \propto \langle\tau\rangle \,.$$

2. Derivation of the PDF $g(\tau)$

The properties that must be satisfied by the stationary correlation function $R(t)$ and by the PDF $g(\tau)$ are listed in the above Box 1.

We now prove the following

Theorem (PDF $g(\tau)$)
Given Eq. (8,MT) defining the stationary correlation function of the Langevin equation with random parameters, Eq. (4,MT), the PDF $g(\tau)$ given in Eq. (14,MT) satisfies all the required constrains (i-iv) listed in Box 1.

Proof:

(i) normalization and (iv) finite mean:
Let us write:

$$g(\tau) = \frac{C}{\tau} L_\eta^{-\eta}\left(\frac{\tau}{\tau_*}\right) \ ,$$

where τ_* must be introduced to get an adimensional parameter as argument of $L_\eta^{-\eta}$. The mean correlation time is given by:

$$\langle\tau\rangle = \int_0^\infty \tau g(\tau) d\tau = C \int_0^\infty L_\eta^{-\eta}\left(\frac{\tau}{\tau_*}\right) d\tau = C\tau_* \ , \tag{27}$$

so that we have:

$$g(\tau) = \frac{C}{\tau} L_\eta^{-\eta}\left(C\frac{\tau}{\langle\tau\rangle}\right) \ . \tag{28}$$

The normalization constant C can be obtained by imposing $\mathcal{L}[g(\tau)](0) = 1$. Exploiting the relationship $\int_s^\infty \exp(-\xi\tau) d\xi = \exp(-\xi\tau)/\tau$ and making the change of variables $\tau = (\langle\tau\rangle/C)\tau'$, we get:

$$\begin{aligned}\mathcal{L}[g(\tau)](s) &= C \cdot \int_{s\langle\tau\rangle/C}^\infty \mathcal{L}[L_\eta^{-\eta}(\tau)](\xi) d\xi \\ &= C \cdot \int_{s\langle\tau\rangle/C}^\infty e^{-\xi^\eta} d\xi = \\ &(x = \xi^\eta) \\ &= C\frac{1}{\eta} \int_{s\langle\tau\rangle/C}^\infty \frac{1}{\eta} e^{-x} x^{1/\eta-1} dx \ ,\end{aligned} \tag{29}$$

and:

$$\begin{aligned}\mathcal{L}[g(\tau)](0) &= C \cdot \int_0^\infty \frac{1}{\eta} e^{-x} x^{1/\eta-1} dx \\ &= C \cdot \frac{\Gamma(1/\eta)}{\eta} = 1 \ .\end{aligned} \tag{30}$$

Substituting this relationship into Eq. (28) we finally get Eq. (14,MT), which is a properly normalized PDF.

(ii) superdiffusive scaling:

We now prove that $R(t) \sim t^{-\eta}$, with $0 < \eta < 1$, a condition leading to the superdiffusive scaling for the position variance: $\sigma_X^2(t) \sim t^{\phi}$, $1 < \phi = 2 - \eta < 2$. This can be proven thanks to the integral representation of the extremal Lévy density:

$$L_\eta^{-\eta}(x) = \frac{1}{\eta x}\frac{1}{2\pi i}\int_{\gamma-i\infty}^{\gamma+i\infty}\frac{\Gamma(s/\eta)}{\Gamma(s)}x^s ds, \quad 0 < \eta < 1\ . \tag{31}$$

Hence, we have:

$$\begin{aligned}
R(t) &= \langle\nu\rangle\frac{\eta}{\Gamma(1/\eta)}\int_0^\infty e^{-t/\tau}L_\eta^{-\eta}\left(\frac{\tau}{\tau_*}\right)d\tau \\
&= \langle\nu\rangle\frac{\eta}{\Gamma(1/\eta)}\int_0^\infty e^{-t/\tau}\left[\frac{1}{\eta}\frac{1}{2\pi i}\int_{\gamma-i\infty}^{\gamma+i\infty}\frac{\Gamma(s/\eta)}{\Gamma(s)}\left(\frac{\tau}{\tau_*}\right)^{(s-1)}ds\right]d\tau \\
&= \langle\nu\rangle\frac{\eta}{\Gamma(1/\eta)}\frac{1}{\eta}\frac{1}{2\pi i}\int_{\gamma-i\infty}^{\gamma+i\infty}\frac{\Gamma(s/\eta)}{\Gamma(s)}\left[\int_0^\infty e^{-t/\tau}\left(\frac{\tau}{\tau_*}\right)^{s-1}d\tau\right]ds = \\
&(\xi = t/\tau) \\
&= \langle\nu\rangle\langle\tau\rangle\frac{1}{\eta}\frac{1}{2\pi i}\int_{\gamma-i\infty}^{\gamma+i\infty}\frac{\Gamma(s/\eta)}{\Gamma(s)}\left[\int_0^\infty e^{-\xi}\xi^{-1-s}\left(\frac{t}{\tau_*}\right)^s d\xi\right]ds \\
&= \langle\nu\rangle\langle\tau\rangle\frac{1}{\eta}\frac{1}{2\pi i}\int_{\gamma-i\infty}^{\gamma+i\infty}\frac{\Gamma(s/\eta)\Gamma(-s)}{\Gamma(s)}\left(\frac{t}{\tau_*}\right)^s ds\ ,
\end{aligned} \tag{32}$$

where $\tau_* = \langle\tau\rangle\,\Gamma(1/\eta)/\eta$. It is useful to rewrite the expression as:

$$\begin{aligned}
R(t) &= \langle\nu\rangle\langle\tau\rangle\frac{1}{\eta}\frac{1}{2\pi i}\int_{\gamma-i\infty}^{\gamma+i\infty}\frac{(\eta/s)\Gamma(s/\eta+1)\Gamma(-s)}{(1/s)\Gamma(s+1)}\left(\frac{t}{\tau_*}\right)^s ds \\
&= \langle\nu\rangle\langle\tau\rangle\frac{1}{2\pi i}\int_{\gamma-i\infty}^{\gamma+i\infty}\frac{\Gamma(s/\eta+1)\Gamma(-s)}{\Gamma(s+1)}\left(\frac{t}{\tau_*}\right)^s ds\ ,
\end{aligned} \tag{33}$$

which can be solved through the residues theorem considering the poles $s/\eta+1 = -n$ or $s = n$, with $n = 0, 1, 2..\infty$.

In the first case we have:

$$\begin{aligned}
R(t) =& \langle\nu\rangle\langle\tau\rangle\sum_{n=0}^{\infty}\eta\frac{(-1)^n}{n!}\frac{\Gamma(\eta(n+1))}{\Gamma(1-\eta(n+1))}\left(\frac{t}{\tau_*}\right)^{-\eta(n+1)} \\
=& \langle\nu\rangle\langle\tau\rangle\sum_{n=1}^{\infty}\frac{(-1)^n}{n!}\frac{\Gamma(\eta n)}{\Gamma(-\eta n)}\left(\frac{t}{\tau_*}\right)^{-\eta n}
\end{aligned} \tag{34}$$

where each term of the series is obtained by the limit:

$$\begin{aligned}
&\lim_{s\to-\eta(n+1)}(s+\eta(n+1))\frac{\Gamma(s/\eta+1)\Gamma(-s)}{\Gamma(s+1)}\left(\frac{t}{\tau_*}\right)^{s}\\
&\lim_{s\to-\eta(n+1)}\eta((s/\eta+1)+n)\frac{\Gamma(s/\eta+1)\Gamma(-s)}{\Gamma(s+1)}\left(\frac{t}{\tau_*}\right)^{s}\\
&\lim_{s\to-\eta(n+1)}\frac{\eta((s/\eta+1)+n)}{(s/\eta+1)_{n+1}}\frac{\Gamma(s/\eta+n+2)\Gamma(-s)}{\Gamma(s+1)}\left(\frac{t}{\tau_*}\right)^{s}\\
&\lim_{s\to-\eta(n+1)}\frac{\eta(-1)^n}{n!}\frac{\Gamma(\eta(n+1))}{\Gamma(1-\eta(n+1))}\left(\frac{t}{\tau_*}\right)^{-\eta(n+1)}
\end{aligned} \tag{35}$$

When $t\to\infty$ only the first term survives and we find:

$$R(t)=\langle\nu\rangle\langle\tau\rangle\frac{\Gamma(\eta+1)}{\Gamma(1-\eta)}\left(\frac{t}{\tau_*}\right)^{-\eta}\quad . \tag{36}$$

Substituting $\tau_*=\langle\tau\rangle\,\Gamma(1/\eta)/\eta$, we finally get Eq. (15,MT), from which we obtain the superdiffusive scaling of the position variance $\sigma_X^2(t)\propto t^{\phi}$, with $\phi=2-\eta$.

Considering the poles in the other semi-plane, $s=n$ with $n=0,1,2..\infty$, we find that:

$$R(t)=\langle\nu\rangle\langle\tau\rangle\frac{1}{\eta}\sum_{n=0}^{\infty}\frac{(-1)^n}{n!}\frac{\Gamma(n/\eta)}{\Gamma(n)}\left(\frac{t}{\tau_*}\right)^{n} \tag{37}$$

converges to $R(0)=\langle\nu\rangle\langle\tau\rangle$, as already shown before.

(iii) MSD at time zero is zero:

The condition $\sigma_X^2(t=0)=0$ is clearly verified.

Example:

In the special case $\eta=1/2$, the extremal Lévy function corresponds to the Lévy–Smirnov distribution, the whole exercise can be solved analitycally and we may consider for simplicity $\langle\tau\rangle\frac{\Gamma(1/\eta)}{\eta}=1$:

$$g(\tau)=\frac{1}{\sqrt{4\pi\tau^5}}e^{-1/(4\tau)} \tag{38}$$

Solving the integral the analytical form of the correlation function turns to be:

$$R(t)=\frac{\Gamma(1/2)}{\sqrt{4\pi}}\left(t+\frac{1}{4}\right)^{-1/2} \tag{39}$$

which leads to the following exact formula for the position variance:

$$\sigma_X^2(t)=\frac{\Gamma(1/2)}{\sqrt{\pi}}\left[\frac{4}{3}\left(t+\frac{1}{4}\right)^{3/2}-t-\frac{1}{6}\right]\ , \tag{40}$$

satisfying both superdiffusive long-time scaling and $\sigma_X^2(0) = 0$ conditions.

NOTE: *The Einstein–Smoluchovsky relation*
By substituting Eq. (12,MT) into Eq. (5) it is easy to see that $\tau_c = \tau$. Using the following equation (see the last equation in Box 1 of the Main Text):

$$R(0|V_0, \tau, \nu) = \langle V^2 | V_0, \tau, \nu \rangle_{\text{st}} = \nu\tau \ ,$$

and substituting Eq. (12,MT) into the definition of D_X, Eq. (8), we get the Einstein–Smoluchowsky relation:

$$D_X = \nu\tau^2 = \tau \ \langle V^2 | V_0, \tau, \nu \rangle_{\text{st}} \ , \tag{41}$$

which, apart from the conditional statistics, is essentially the same as Eq. (9). For a standard OU process with fixed ν and τ, $\langle V^2 | V_0, \tau, \nu \rangle_{\text{st}} = \langle V^2 \rangle_{\text{eq}}$ and Eq. (41) relates the diffusion (D_X) and relaxation (τ) properties through the equilibrium distribution ($\langle V^2 \rangle_{\text{eq}}$). In his 1905 paper [2], Einstein studied the Brownian motion in a gas at equilibrium, where velocity distribution is given by the Maxwell–Boltzmann law. In this case, the Einstein–Smoluchowsky relation becomes:

$$D_X = \tau \ \langle V^2 \rangle_{\text{st}} = \tau \ \frac{kT}{m} \ , \tag{42}$$

being T, m and k the gas temperature, the Brownian particle mass and the Boltzmann constant, respectively.

3. Numerical scheme for the Langevin equation

In order to avoid stability problems, the numerical algorithm for the simulation of Eqs. (1) and 4,MT) was implemented using an implicit scheme with order of strong convergence 1.5 [6]. This is given by the following expression:

$$\begin{aligned} V_{n+1} = V_n + b\Delta W_n \quad &+ \quad \frac{1}{2}\{a(V_{n+1}) + a(V_n)\} + \\ &+ \quad \frac{1}{2\sqrt{\Delta t}}\{a(\overline{V}_+) - a(\overline{V}_-)\}\left(\Delta Z_n - \frac{1}{2}\Delta W_n \Delta t\right) \ , \end{aligned} \tag{43}$$

being $V_n = V(n\Delta t)$, Δt the time step, $\Delta W_n = W(t_n + \Delta t) - W(t_n)$ the increments of the Wiener process, $a(V) = -V/\tau$ and $b = \sqrt{2\nu}$ the drift and noise terms, respectively. Further, we have:

$$\begin{aligned} \overline{V}_\pm &= V_n + a(V_n)\Delta t \pm b\sqrt{\Delta t} \,, \\ \Delta Z_n &= \frac{1}{2}(\Delta t)^{3/2}\left(u_1(n) + \frac{1}{\sqrt{3}}u_2(n)\right) \ , \end{aligned} \tag{44}$$

being $u_1(n)$ and $u_2(n)$ two independent random numbers with uniform distributions in $[0, 1]$. A suitable time step Δt, also depending on the time scale τ, is necessary to maintain the accuracy of the numerical scheme. To take into account both the ensemble variability of the relaxation time τ, which is different

for different trajectories, and the time variability of drift and noise terms along the same trajectory, we applied a variable time step according to the scheme given in Ref. [7]:

$$\Delta t = \min\left\{\frac{0.05}{b}, \frac{0.1}{|a|}\right\} . \tag{45}$$

This adaptive time step allows to avoid any problem of convergence and accuracy in the numerical scheme, Eqs. (43) and (44). At the same time, in the range of short τ, this algorithm can give very short time steps, thus determining very long simulation times for a consistent number of trajectories. To overcome this problem we note that the short time regime $\tau \ll \langle\tau\rangle$ of the PDF $g(\tau)$ does not significantly affect the anomalous scaling of diffusion, which mostly depends on the asymptotic tail of the distribution $g(\tau)$. A cut-off was then introduced in the short-time regime. By comparing the numerical simulations with theoretical results we chose the cut-off value $\tau_{\min} = 0.004$, much smaller that $\langle\tau\rangle$, which is always of the order $0.5 - 1$ for all sampled sets of τ.

4. Numerical algorithm for the random generator of τ

Here we describe a method to generate random variables τ distributed according to the law of Eq. (14,MT),

$$g(\tau) = A(\eta) L_\eta^{-\eta}(\tau)/\tau, \tag{46}$$

where $A(\eta)$ is the normalization coefficient, and τ is already dimensionless.

For this, we use a well-known inverse transform sampling method (see, e.g. [8]), so the procedure is straightforward.

First, we generate a set of extremal Lévy density random numbers $L_\eta^{-\eta}(\tau)$ by using the generator described in Refs. [9, 10], see Eq. (3.2) of the latter paper, and extract its histogram. Since the beginning of the histogram has much statistical noise (red curve in Fig. 2a), it is a good solution to replace these values with analytical asymptote at small arguments [11] (blue curve in Fig. 2a). Moreover, we also expand the histogram with another asymptote, at large τs (green curve in Fig. 2a):

$$L_\eta^{-\eta}(\tau) \quad \sim \quad A_1 \tau^{-a_1} \exp(-b_1 \tau^{c_1}), \qquad \tau \to 0^+, \tag{47}$$

$$L_\eta^{-\eta}(\tau) \quad \sim \quad \frac{C_1(\eta)}{|\tau|^{1+\eta}}, \qquad \tau \to \infty, \tag{48}$$

where

$$A_1 = \left\{[2\pi(1-\eta)]^{-1}\, \eta^{1/(1-\eta)}\right\}^{1/2}, \tag{49}$$

$$a_1 = \frac{2-\eta}{2(1-\eta)}, \quad b_1 = (1-\eta)\eta^{\eta/(1-\eta)}, \quad c_1 = \frac{\eta}{1-\eta} \tag{50}$$

$$C_1(\eta) \approx \frac{1}{\pi} \sin\left(\pi \frac{\eta}{2}\right) \Gamma(1+\eta). \tag{51}$$

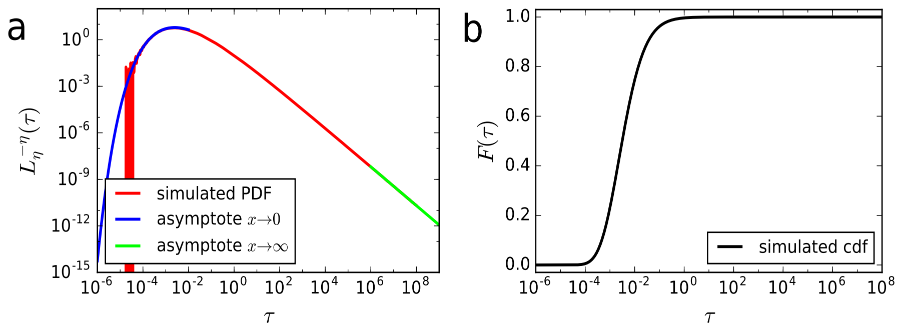

Figure 2: (color online) (a) Simulated Lévy extremal density (red) together with asymptotics at small arguments (blue) and large ones (green). (b) Cumulative distribution function of $g(\tau)$.

Then, we divide the obtained histogram by argument and find the normalization coefficient numerically in order that the resulting PDF is normalized to unity. Finally, we calculate the semi-analytical cumulative distribution function (CDF) (see Fig. 2):

$$F(\tau_k) = \sum_{i=0}^{k} g(\tau_i)\delta\tau_i, \qquad \tau_k \le \tau_n; \tag{52}$$

$$F(\tau) = \sum_{i=0}^{n} g(\tau_i)\delta\tau_i + \int_{\tau_n}^{\tau} \frac{A(\eta)}{\tau'^{2+\eta}} d\tau', \qquad \tau > \tau_n, \tag{53}$$

where $\delta\tau_i$ is the ith histogram's bin width, $i = 0, 1, 2..n$.

Now, we draw a random variable τ obeying the target pdf (46) with

$$\tau = F^{-1}(u), \tag{54}$$

where $u \in [0, 1)$ is a uniformly distributed random variable: F^{-1} is a numerically (or if $u > F(x_n)$, semi-analytically) inverted CDF.

Let us take out a verification and compare the original PDF $g(\tau)$ used for the simulations and the histogram of the generated 10^7 random numbers with this algorithm $g_{\text{sim}}(\tau)$. The result is shown in Fig. 3. At intermediate values of τ the inaccuracy is about 1%, increasing due to statistical error at very small and large τs (where $g(\tau)$ is small).

The software for the numerical simulations were written in C++ language (Debian gcc 4.9) and Python 2.7 and can be downloaded at the following web-site: https://gitlab.bcamath.org/opensource/lecm.
The codes include the algorithms described in this section and in the previous one. The simulation runs were performed on computational facilities of BCAM-Basque Center for Applied Mathematics.

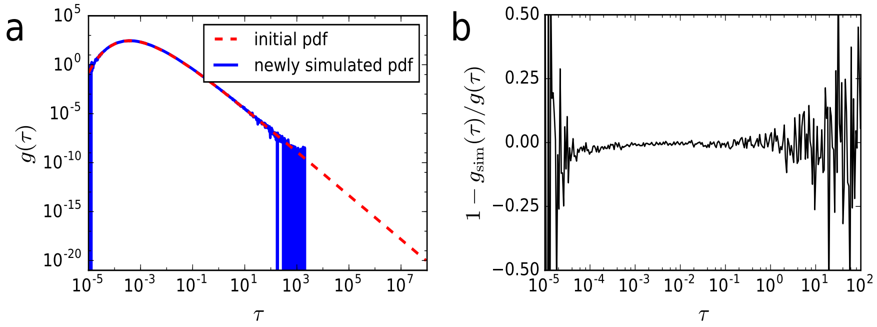

Figure 3: (color online) (a) Comparison of the original PDF (46) (red) and the PDF histogram of generated numbers (blue). (b) Relative error between original and simulated PDFs.

5. Schneider grey noise, gBM and ggBM

We here provide an intuitive presentation of the Schneider grey noise, the grey Brownian motion and the generalized grey Brownian motion. More rigouros details can be found in [12, 13, 14, 15, 16, 17, 18, 19].

The grey noise is a generalization on the basis of the Mittag–Leffler function of the white noise. The Mittag-Leffler function $E_\beta(z)$ is defined as

$$E_\beta(z) = \sum_{n=0}^{\infty} \frac{z^n}{\Gamma(\beta n + 1)} \,, \tag{55}$$

and it is a generalization of the exponential function that is recovered as special case when $\beta = 1$, i.e., $E_1(-z) = \mathrm{e}^{-z}$. As well as the exponential function, when $0 < \beta < 1$, the Mittag–Leffler function is a completely monotonic function. A useful formula for what follows is

$$-\frac{d^2}{dz^2} E_\beta(-z^2\, q)\Big|_{z=0} = \frac{2}{\Gamma(1+\beta)}\, q \,. \tag{56}$$

For any characteristic functional $\Phi(z)$ there exists a unique probability measure μ such that

$$\Phi(z) = \int_{-\infty}^{+\infty} \mathrm{e}^{iz\tau}\, d\mu(\tau) \,, \tag{57}$$

and if $\Phi(z) = E_\beta(-z^2)$, $0 < \beta < 1$, the probability measure μ is the so-called Schneider grey noise [12, 13, 17]. When $\beta = 1$ we have $E_1(-z^2) = \mathrm{e}^{-z^2}$, and the Gaussian white noise follows.

Let us introduce the stochastic process $X(t)$ driven by the noise μ and we look for its probability density function. The characteristic function is

$$\langle \mathrm{e}^{izX(t)} \rangle = \int_{-\infty}^{+\infty} \mathrm{e}^{izX(t)}\, d\mu(t) = E_\beta(-z^2\, \varphi_\alpha^2(t)) \,, \tag{58}$$

where function $\varphi_\alpha(t)$ takes into account what remains of parameter t after the integration, and it is related to the scaling in time of $X(t)$. By the inversion of (58) we have the probability density function of $X(t)$ as follows

$$p(x,t) = \frac{1}{2\pi}\int_{-\infty}^{+\infty} \mathrm{e}^{-izx} E_\beta(-z^2\,\varphi_\alpha^2(t))dz = \frac{1}{2\,\varphi_\alpha(t)} M_{\beta/2}\left(\frac{|x|}{\varphi_\alpha(t)}\right) , \tag{59}$$

where $M_{\beta/2}$ is the M-Wrigth/Mainardi function. By using (58) and (56), we have that the variance of $X(t)$ is

$$\langle x^2 \rangle = -\frac{d^2}{dz^2} E_\beta(-z^2\,\varphi_\alpha^2(t))\Big|_{z=0} = \frac{2}{\Gamma(1+\beta)}\varphi_\alpha^2(t)\,. \tag{60}$$

In the same spirit, the correlation function of the process $X(t)$ can be computed. In fact from (58) it holds

$$\langle \mathrm{e}^{iz[X(t)-X(s)]}\rangle = \int_{-\infty}^{+\infty} \mathrm{e}^{iz[X(t)-X(s)]}\, d\mu(t,s) = E_\beta(-z^2\,\varphi_\alpha^2(t,s))\,, \tag{61}$$

and by applying again formula (56) the correlation function results to be

$$\frac{1}{\Gamma(1+\beta)}\,(\varphi_\alpha(t) + \varphi_\alpha(s) - \varphi_\alpha(t,s))\,. \tag{62}$$

Now we discuss how to establish function $\varphi_\alpha(t)$. Let $\mathbb{1}_{[a,b]}$ be the indicator function such that it is equal to 1 when $a < t < b$ and to 0 elsewhere. In analogy with the Wiener process where the Brownian motion is $\mathcal{B}(t) = \int_0^t dW(\tau)$, we write the process $X(t)$ as

$$X(t) = \int_0^t d\mu(\tau) = \mathbb{1}_{[0,t]}\, X_0(\mathbb{1}_{[0,t]})\,, \tag{63}$$

where X_0 is a random variable equivalent in distribution to $X(t)$ but independent of t, i.e., the probability density function of X_0 is $p_0(x) = p(x, t = 1)$. From (63) we have that

$$\langle [X(t)]^2\rangle = \langle [\mathbb{1}_{[0,t]}]^2\rangle\, \langle [X_0]^2\rangle\,, \tag{64}$$

and from comparison with (60) and (62), we obtain that $\varphi_\alpha(t)$ is established through the stochastic process $\mathbb{1}_{[a,b]}$ that meets

$$\langle [\mathbb{1}_{[0,t]}]^2\rangle = \varphi_\alpha^2(t)\,, \tag{65}$$

$$\langle \mathbb{1}_{[s,t]}\mathbb{1}_{[0,s]}\rangle = \frac{1}{2}(\varphi_\alpha^2(t) + \varphi_\alpha^2(s) - \varphi_\alpha^2(t,s))\,. \tag{66}$$

Finally we observe that, by setting $\varphi_\alpha^2(t) = t^\alpha$, $X(t)$ is the Brownian motion when $\alpha = \beta = 1$, and we refer to it as the grey Brownian motion and the

generalized grey Brownian motion when $0 < \alpha = \beta < 1$ and $0 < \alpha < 2$, $0 < \beta < 1$, respectively. Moreover, in order to have a process with stationary increments we assume $\varphi^2_\alpha(t,s) = |t-s|^\alpha$, the correlation function results to be

$$\frac{1}{\Gamma(1+\beta)}\left(t^\alpha + s^\alpha - |t-s|^\alpha\right). \tag{67}$$

The corresponding stochastic process is obtained with a randomly-scaled Gaussian process, i.e., a Gaussian process multiplied for a non-negative independent randon variable not dependent on time.

From integral representation formulae of the M function [20], we have that X_0 has the same density of $X(1)$ if, for example, we state $X_0 = \sqrt{\Lambda}\,\mathcal{B}(1)$ where Λ is a non-negative random variable distributed according to M_β and $\mathcal{B}(1)$ is a Gaussian variable. Finally, we obtain that

$$X(t) = \sqrt{\Lambda}\ \mathbb{1}_{[0,t]}\,\mathcal{B}(\mathbb{1}_{[0,t]}). \tag{68}$$

Looking at (60) and (62), the process $\mathbb{1}_{[0,t]}\,\mathcal{B}(\mathbb{1}_{[0,t]})$ is the fractional Brownian motion $X_H(t)$ [21] characterized by

$$\langle [X_H(t)]^2\rangle = t^{2H}, \tag{69}$$

$$\langle X(t)X(s)\rangle = \frac{1}{2}(t^{2H} + s^{2H} - |t-s|^{2H}). \tag{70}$$

Finally, by setting $H = \alpha/2$, the trajectories of the process $X(t)$ can be generated by

$$X(t) = \sqrt{\Lambda}\,X_H(t). \tag{71}$$

Since the fBm $X_H(t)$ is fully characterized by the variance and the correlation functio, the process $X(t)$ is also fully characterized by the variance and the correlation function.
With a somewhat forced terminology, the term ggBM can be thought to include any randomly scaled Gaussian process, i.e., any processes defined by the product of a Gaussian process with an independent and constant non-negative random variable.

6. Mainardi distribution and Lévy densities

Fractional diffusion processes are a generalization of classical Gaussian diffusion, mainly in the direction of the time-fractional diffusion, i.e., by replacing the first derivative in time with a time-fractional derivative, and in the direction of the space-fractional diffusion, i.e., by replacing the second derivative in space with a space-fractional derivative. In the case of time-fractional diffusion the Gaussian particle density is generalized by the so-called M-Wright/Mainardi functions [22, 23], and in the case of the space-fractional diffusion the particle density is generalized by the so-called Lévy stable densities [11].

The M-Wright/Mainardi function $M_\nu(r)$, $r \geq 0$, $0 < \nu < 1$, is defined by the series:

$$M_\nu(r) = \sum_{n=0}^{\infty} \frac{(-r)^n}{n!\Gamma[-\nu n + (1-\nu)]} = \frac{1}{\pi} \sum_{n=0}^{\infty} \frac{(-r)^{n-1}}{(n-1)!} \Gamma(\nu n) \sin(\pi \nu n) \,, \tag{72}$$

and it provides a generalization of the Gaussian and Airy functions:

$$M_{1/2}(r) = \frac{1}{\sqrt{\pi}} \, \mathrm{e}^{-r^2/4} \,, \quad M_{1/3}(r) = 3^{2/3} Ai(r/3^{1/3}) \,. \tag{73}$$

Moreover, the following limit holds:

$$\lim_{\nu \to 1^-} M_\nu(r) = \delta(r-1) \,. \tag{74}$$

The M density function is related to the Mittag–Leffler function through the Laplace transform:

$$\int_0^\infty \mathrm{e}^{-\lambda r} M_\nu(r) \, dr = E_\nu(-\lambda) \,, \tag{75}$$

and it has an exponential decay for $r \to \infty$, i.e.:

$$M_\nu(r) \sim \frac{Y^{\nu-1/2}}{\sqrt{2\pi}(1-\nu)^\nu \nu^{2\nu-1}} \, \mathrm{e}^{-Y} \,, \quad Y = (1-\nu)(\nu^\nu r)^{1/(1-\nu)} \,, \tag{76}$$

which allows for finite moments that can be computed through the formula:

$$\int_0^\infty r^q M_\nu(r) dr = \frac{\Gamma(q+1)}{\Gamma(\nu q + 1)} \,, \quad q > -1 \,. \tag{77}$$

A remarkable formula of the Mainardi density is the following integral representation with $r \geq 0$, $0 < \nu \,, \eta \,, \beta < 1$ [20]:

$$M_\nu(r) = \int_0^\infty M_\eta \left(\frac{r}{\tau^\eta} \right) M_\beta(\tau) \, \frac{d\tau}{\tau^\eta} \,; \quad \nu = \eta\beta \,, \tag{78}$$

that, in the special case $\eta = 1/2$, provides the following link with the Gaussian density:

$$M_{\beta/2}(r) = \int_0^\infty \frac{\mathrm{e}^{-r^2/(4\tau)}}{\sqrt{\pi\tau}} M_\beta(\tau) \, \frac{d\tau}{\tau^\eta} \,. \tag{79}$$

The Lévy stable density $L_\alpha^\theta(z)$, $-\infty < z < +\infty$, $0 < \alpha < 2$, $|\theta| = \min\{\alpha, 2-\alpha\}$, is defined through the Fourier transform:

$$\int_{-\infty}^{+\infty} \mathrm{e}^{i\kappa z} L_\alpha^\theta(z) d\kappa = \mathrm{e}^{-\Psi(\kappa)} \,, \quad \Psi(\kappa) = |\kappa|^\alpha \mathrm{e}^{i(\mathrm{sgn}\,\kappa)\theta\pi/2} \,. \tag{80}$$

In the case $\theta = -\alpha$, $0 < \alpha < 1$, the Lévy density reduces to a one-side density on the positive semi-axis (when $\theta = \alpha$ on the negative semi-axis) and it is defined through the Laplace transform:

$$\int_0^\infty \mathrm{e}^{sz} L_\alpha^{-\alpha}(z) dz = \mathrm{e}^{-s^\alpha} \,. \tag{81}$$

The asymptotic behaviour for $|z| \to \infty$ is the power-law

$$L_\alpha^\theta(z) = \mathcal{O}(|z|^{-(\alpha+1)}\,, \tag{82}$$

and, for extremal densities, the following exponential decay holds for $z \to 0$:

$$L_\alpha^{-\alpha}(z) \sim \frac{z^{-(2-\alpha)/(2(1-\alpha))}}{\sqrt{2\pi(1-\alpha)\alpha^{1/(\alpha-1)}}}\,\mathrm{e}^{-Y}\,, \quad Y = (1-\alpha)\alpha^{\alpha/(1-\alpha)} z^{\alpha/(1-\alpha)}\,. \tag{83}$$

Important special cases are the Gaussian, the Cauchy and the Lévy–Smirnov density, i.e.:

$$L_2^0(z) = \frac{\mathrm{e}^{-z^2/4}}{2\sqrt{\pi}}\,, \quad L_1^0(z) = \frac{1}{\pi}\frac{1}{1+z^2}\,, \quad L_{1/2}^{-1/2}(z) = \frac{z^{-3/2}}{2\sqrt{\pi}}\,\mathrm{e}^{-1/(4z)}\,. \tag{84}$$

Moreover, the following limit holds:

$$\lim_{\alpha\to 1} L_\alpha^{-\alpha}(z) = \delta(z-1)\,. \tag{85}$$

A remarkable formula of the Lévy density is the following integral representation for $z \ge 0$, $0 < \beta < 1$:

$$L_{\alpha_p}^{\theta_p}(z) = \int_0^\infty L_{\alpha_q}^{\theta_q}\left(\frac{z}{\tau^{1/\theta_q}}\right) L_\beta^{-\beta}(\tau)\frac{d\tau}{\tau^{1/\alpha_q}}\,, \quad \alpha_p = \beta\alpha_q\,, \quad \theta_p = \beta\theta_q\,, \tag{86}$$

that, in the special case $\alpha_q = 2$, $\theta_q = 0$, provides the following link with the Gaussian density [20, 24]:

$$L_\alpha^0(z) = \int_0^\infty \frac{\mathrm{e}^{-z^2/(4\tau)}}{\sqrt{\pi\tau}} L_{\alpha/2}^{-\alpha/2}(\tau)d\tau\,. \tag{87}$$

The $M_\nu(r)$ function, $r \ge 0$, $0 < \nu < 1$, and the extremal Lévy density $L_\nu^{-\nu}(r)$ are related by the formula:

$$\frac{1}{c^{1/\nu}} L_\nu^{-\nu}\left(\frac{r}{c^{1/\nu}}\right) = \frac{c\,\nu}{r^{\nu+1}} M_\nu\left(\frac{c}{r^\nu}\right)\,, \quad c > 0\,. \tag{88}$$

In the present paper we consider such special densities in order to highlight the relation of the proposed formulation with the fractional diffusion. However, the asympototic behaviour of the modeled diffusion can be achieved by using the asymptotic behaviour of the involved densities. This means, by using exponential and power-law functions rather than special functions.

7. Space-Time Fractional Diffusion

For the particular choice of parameters: $\phi = 2\beta/\alpha$; $1 < \phi < 2$, Eq. (20,MT) reduces to the fundamental solution of the following Space-Time Fractional Diffusion equation:

$$_tD_*^\beta\, p(x;t) = A_\alpha\; {_xD_0^\alpha}\, p(x;t)\,, \quad -\infty < x < +\infty\,, \quad t \ge 0\,, \tag{89}$$

with:

$$A_\alpha = (C\overline{\nu})^{\alpha/2} \; . \tag{90}$$

The nonlocal operators ${}_tD_*^\beta$ and ${}_xD_0^\alpha$ are the Caputo fractional time derivative and the Riesz-Feller space derivative, respectively (see [11] for the definition of these operators). This is the same equation discussed in Refs. [11, 25], but with a generalized fractional diffusivity A_α different from 1.

The solution reads:

$$K_{\alpha,\beta}^\theta(x,t) = \frac{1}{(A_\alpha)^{1/\alpha}\, t^{\beta/\alpha}} K_{\alpha,\beta}^\theta\left(\frac{x}{(A_\alpha)^{1/\alpha}\, t^{\beta/\alpha}}\right) ,$$

with $\theta = 0$ in this case. [6]. The superdiffusive regime determines the following constrain on α and β: $\alpha/2 < \beta < \alpha$.

Given the solutions of the Time Fractional Diffusion equation and of the Space Fractional Diffusion equation with diffusivity 1 and A_α, respectively [11, 26]:
$M_\beta(x,t) = 1/t^\beta M_\beta(x/t^\beta)$ (Mainardi probability density) and
$L_\alpha^\theta(x,t) = 1/\left(A_\alpha t\right)^{1/\alpha} L_\alpha^\theta\left(x/\left(A_\alpha t\right)^{1/\alpha}\right)$ (Lévy probability density),
the general solution $K_{\alpha,\beta}^\theta$ can be written as a combination of these same solutions:

$$K_{\alpha,\beta}^\theta(x,t) = \int_0^\infty L_\alpha^\theta(x,\tau) M_\beta\left(\tau,t\right)\, d\tau \, , \tag{91}$$

then the general solution emerges as a linear combination of the temporal (Mainardi) and spatial (Lévy) solutions. The Mainardi density is related to the extremal Lévy density by the following relationship (see Section 6 for details):

$$\frac{t}{\beta\tau}\frac{1}{\tau^{1/\beta}} L_\beta^{-\beta}\left(\frac{t}{\tau^{1/\beta}}\right) = \frac{1}{t^\beta}\, M_\beta\left(\frac{\tau}{t^\beta}\right) \, , \quad 0 < \beta \le 1 \, , \quad \tau, t \ge 0 \, , \tag{92}$$

References

[1] G. I. Taylor, Diffusion by continuos movements, Proc. London Math. Soc. s2-20 (1) (1921) 196–211. arXiv:http://plms.oxfordjournals.org/content/s2-20/1/196.full.pdf+html, doi:10.1112/plms/s2-20.1.196.
URL http://plms.oxfordjournals.org/content/s2-20/1/196.short

[6] Due to the self-similar property, here and in the following we use the same symbol for the two-variable function $F(x,t)$ and the associated one-variable function written in terms of the similarity variable. Then, given the scaling exponent Λ and the coefficient A, we write: $F(x,t) = 1/t^\Lambda F(x/(At^\Lambda))$. This notation is not ambiguous as the meaning clearly follows from the number of independent variables.

[2] A. Einstein, Uber die von der molekularkinetischen theorie der warme geforderte bewegung von in ruhenden flussigkeiten suspendierten teilchen, Annalen der Physik 322 (8) (1905) 549–560.

[3] B. B. Mandelbrot, J. W. Van Ness, Fractional brownian motions, fractional noises and applications, SIAM Rev. 10 (4) (1968) 422–437.

[4] T. Dieker, Simulation of fractional Brownian motion, 2004, ph.D. Thesis, Physics Department of Mathematical Sciences, University of Twente. URL http://www.columbia.edu/~ad3217/fbm/thesis.pdf

[5] W. Feller, An Introduction to Probability Theory and its Applications, 2nd Edition, Vol. 2, Wiley, New York, 1971.

[6] P. E. Kloeden, E. Platen, Numerical solution of Stochastic Differential Equations, Springer-Verlag, 1992.

[7] D. J. Thomson, Criteria for the selection of stochastic models of particle trajectories in turbulent flows, J. Fluid Mech. 180 (1987) 529–556.

[8] L. Devroye, Nonuniform random variate generation, Handbooks in operations research and management science 13 (2006) 83–121.

[9] J. M. Chambers, C. L. Mallows, B. W. Stuck, A method for simulating skewed stable random variables, J. Amer. Statist. Assoc. 71 (1976) 340–344.

[10] R. Weron, On the Chambers–Mallows–Stuck method for simulating skewed stable random variables, Statist. Probab. Lett. 28 (1996) 165–171, corrigendum: http://mpra.ub.uni-muenchen.de/20761/1/RWeron96_Corr.pdf or http://www.im.pwr.wroc.pl/∼hugo/RePEc/wuu/wpaper/HSC_96_01.pdf.

[11] F. Mainardi, Y. Luchko, G. Pagnini, The fundamental solution of the space-time fractional diffusion equation, Fract. Calc. Appl. Anal. 4 (2) (2001) 153–192.

[12] W. R. Schneider, Grey noise, in: S. Albeverio, G. Casati, U. Cattaneo, D. Merlini, R. Moresi (Eds.), Stochastic processes, physics and geometry, World Sci. Publ., Singapore, 1990, pp. 676–681.

[13] W. Schneider, Grey noise, in: S. Albeverio, J. E. Fenstad, H. Holden, T. Lindstrøm (Eds.), Ideas and methods in mathematical analysis, stochastics, and applications (Oslo, 1988), Vol. I, Cambridge Univ. Press, Cambridge, 1990, pp. 261–282.

[14] A. Mura, Non-Markovian Stochastic Processes and Their Applications: From Anomalous Diffusion to Time Series Analysis, Lambert Academic Publishing, 2011, ph.D. Thesis, Physics Department, University of Bologna, 2008.

[15] A. Mura, G. Pagnini, Characterizations and simulations of a class of stochastic processes to model anomalous diffusion, J. Phys. A: Math. Theor. 41 (2008) 285003.

[16] A. Mura, M. Taqqu, F. Mainardi, Non-Markovian diffusion equations and processes: Analysis and simulations, Physica A 387 (2008) 5033–5064.

[17] A. Mura, F. Mainardi, A class of self-similar stochastic processes with stationary increments to model anomalous diffusion in physics, Integr. Transf. Spec. F. 20 (3–4) (2009) 185–198.

[18] M. Grothaus, F. Jahnert, F. Riemann, J. da Silva, Mittag–Leffler analysis I: Construction and characterization, J. Funct. Anal. 268 (2015) 1876–1903.

[19] M. Grothaus, F. Jahnert, Mittag–Leffler analysis II: Application to the fractional heat equation, J. Funct. Anal. 270 (2016) 2732–2768.

[20] F. Mainardi, G. Pagnini, R. Gorenflo, Mellin transform and subordination laws in fractional diffusion processes, Fract. Calc. Appl. Anal. 6 (4) (2003) 441–459.

[21] F. Biagini, Y. Hu, B. Øksendal, T. Zhang, Stochastic Calculus for Fractional Brownian Motion and Applications, Springer, 2008.

[22] F. Mainardi, A. Mura, G. Pagnini, The M-Wright function in time-fractional diffusion processes: A tutorial survey, Int. J. Differ. Equations 2010 (2010) 104505.

[23] G. Pagnini, The M-Wright function as a generalization of the Gaussian density for fractional diffusion processes, Fract. Calc. Appl. Anal. 16 (2) (2013) 436–453.

[24] F. Mainardi, G. Pagnini, R. Gorenflo, Mellin convolution for subordinated stable processes, J. Math. Sci. 132 (5) (2006) 637–642.

[25] G. Pagnini, P. Paradisi, A stochastic solution with Gaussian stationary increments of the symmetric space-time fractional diffusion equation, Fract. Calc. Appl. Anal. 19 (2) (2016) 408–440.

[26] R. Gorenflo, F. Mainardi, D. Moretti, G. Pagnini, P. Paradisi, Discrete random walk models for space-time fractional diffusion, Chem. Phys. 284 (2002) 521–541.
